# Physical Activity, Boredom and Fear of COVID-19 Among Adolescents in Germany

**DOI:** 10.3389/fpsyg.2021.624206

**Published:** 2021-05-03

**Authors:** Vincent Bösselmann, Sandra Amatriain-Fernández, Thomas Gronwald, Eric Murillo-Rodríguez, Sergio Machado, Henning Budde

**Affiliations:** ^1^Faculty of Human Sciences, MSH Medical School Hamburg, Hamburg, Germany; ^2^Department of Performance, Neuroscience, Therapy and Health, Faculty of Health Sciences, MSH Medical School Hamburg, Hamburg, Germany; ^3^División Ciencias de la Salud, Escuela de Medicina, Universidad Anáhuac Mayab, Mérida, Mexico; ^4^Laboratory of Physical Activity Neuroscience, Physical Activity Sciences Postgraduate Program, Salgado de Oliveira University, Niterói, Brazil; ^5^Intercontinental Neuroscience Research Group, Mérida, Mexico

**Keywords:** physical activity, physical exercise, boredom, fear, anxiety, COVID-19, adolescents

## Abstract

**Background:**

The effectiveness of physical activity (PA) as an intervention against anxiety disorders and depression is undeniable in clinical psychology. Therefore, the question arose whether these effects also occur when a fear stimulus, like the COVID-19 pandemic, affects otherwise healthy adolescents. Boredom is closely linked to symptoms of fear and anxiety, but the connection between PA, boredom and fear is partly unclear.

**Methods:**

A cross-sectional online study was conducted that involved 122 students. Participants were 13–19 years old (*M*_*age*_ = 15.83, *SD* = 1.73). The survey was available online from April 27th to May 3rd, 2020. At this time, schools in Germany had already been closed for 6 weeks. A self-report questionnaire was used to measure physical activity, boredom, and fear of COVID-19. A multiple linear regression model was conducted.

**Results:**

The reported fear of COVID-19 significantly correlates with total PA, quantity of strenuous PA, and boredom. Furthermore, a significant regression equation was found. The variables boredom, PA, and age contribute significantly to predicting the fear of COVID-19 [*R*^2^ = 0.127, *F*(3,118) = 6.876, *p* < 0.000], among adolescents.

**Conclusion:**

Our results indicate that there is an association between PA, boredom and the quarantine experience of adolescents. Students who were physically more active, especially with strenuous intensity, did not feel bored and showed less fear of COVID-19.

## Introduction

On January 30th the World Health Organization (WHO) classified the Coronavirus as a “public health emergency of international concern” (World Health Organization, 2020c), p1. On March 11th, the WHO said that COVID-19 “can be characterized as a pandemic” (World Health Organization, 2020a), p1. As a reaction to the exacerbating situation and the WHO’s warnings, many countries decided to introduce public life restrictions. On March 22nd, 21.463 cases of COVID-19 were counted in Germany by the WHO. The German government, therefore, decided to introduce a contact lock for more than two persons and several restrictions to the public life, such as a minimum distance of 1.5 m between people in public spaces and the closure of public facilities, like kindergartens and schools (World Health Organization, 2020b). On April 28th, the German society had been living with the corona safeguard measures for 6 weeks. It is difficult, to describe the dynamic development of the pandemic accurately, but it may give an idea of how much stress the pandemic has caused for a significant number of individuals. Not surprisingly, researchers found that many people report an increased feeling of fear in quarantine (Reynolds et al., 2007). Previous studies have shown that even a few-day period of isolation can cause severe consequences to society, such as post-traumatic stress symptoms, anger, and the fear of infection (Brooks et al., 2020). Moreover, boredom, frustration and separation from others are included in the serious consequences of isolation as well and they should not be underestimated (Cava et al., 2005). Students seem to be particularly sensitive to the effects of a pandemic, as they are mainly active in institutional structures (e.g., schools, clubs) and do not have experience in self-organizing their days, which leads to extended sedentary activities (Brazendale et al., 2017). Additionally, quarantine and school cancelation inevitably lead to a reduction of physical activity (Amatriain-Fernández et al., 2020b). In the time of the lockdown students did not have guided physical education lessons or organized free-time activities anymore, playgrounds and outdoor sports facilities were forbidden to be used, and many young people were not allowed to spend time outside their home due to their parent’s fear of them contracting the virus.

Since this research is investigating physical activity and in order to avoid confusion of terms, the difference between physical activity (PA) and physical exercise (PE) should be clarified (Budde et al., 2016a). PA is defined as any bodily movement produced by the contraction of skeletal muscles that significantly boosts the calorie consumption compared to resting energy expenditure (American College of Sports Medicine, Riebe et al., 2018). PE can be defined as one subcategory of PA and mainly differs from PA in its planned and structured execution, and the objective to improve or maintain one’s physical fitness domains (Caspersen et al., 1985). PE is always included in the category of PA, however, the conception of PA is wider than PE (Wegner et al., 2020). Our study aimed to measure (total) PA within 1 week, including easy walking, leisure activities, and PE. The German “national recommendation for exercise and physical activity promotion” recommends 90 min of daily moderate to vigorous PA to adolescents from 12 to 18 years (Pfeifer et al., 2016). Due to the current situation, it can be assumed that most people were unable to accomplish this amount of PA.

The terms fear and anxiety are deliberately used synonymously by some scientists. However, many research results suggest that fear and anxiety differ and are two distinct emotions (Sylvers et al., 2011). According to McNaughton and Corr (2004), fear is present when actively leaving a dangerous situation (active avoidance), and anxiety operates when a dangerous situation is entered or approached (passive avoidance). In this study a fear questionnaire originally developed to measure the fear of Severe Acute Respiratory Syndrome (SARS) was used, since no fear questionnaire for COVID-19 was available at the time of the data collection (Ho et al., 2005). The questionnaire contains items on the active fear of the virus (fear that I will be infected) and items on the underlying mood in an epidemic situation (SARS makes me feel that life is threatening). Thus, it does not seem possible to speak of a very selective recording of fear in the questionnaire used, as both active and passive avoidance behavior are measured.

We chose adolescent students as the sample of interest, since they were unable to follow their daily routine during the lockdown, which could make them extraordinarily vulnerable to this situation (Dubey et al., 2020). The main idea of this study was to investigate the effects of PA on the fear during the COVID-19-pandemic. Since research has shown that boredom and symptoms of anxiety are connected (Lee and Zelman, 2019), we decided to also collect data of boredom to further investigate the connection between PA, boredom, and fear of COVID-19 (FOC-19). In terms of PA studies have shown that participation in sports contributes to a greater well-being of adolescents (McMahon et al., 2016). In contrast, physical inactivity is significantly related to symptoms of anxiety and depression (Bélair et al., 2018). Therefore, we hypothesized that participants with higher PA scores would have less fear, higher boredom scores would correlate with higher FOC-19 scores, and that higher PA scores would also indicate lower boredom scores.

## Materials and Methods

### Participants

The study involved 122 students [69 females (56.6%), 53 males (43.4%)], ages range from 13 to 19 (*M*_*age*_ = 15.83, *SD* = 1.73). All of the participants attended a school in the German federal states Hamburg or Schleswig-Holstein. Participants, who have attended a vocational school and accept vocational training, have been excluded from the study.

### Ethics

All participants (or their legal guardian) provided their written informed consent to participate in this study and have been informed about data protection and use at the start of the questionnaire. They were also informed that they could withdraw from the study at any time without consequences. The study was approved by the Ethics Commission of the MSH Medical School Hamburg (Germany) and conducted in accordance with the Declaration of Helsinki (World Medical Association, 2018).

### Measures and Procedures

For our cross-sectional survey, economic measuring instruments were sought as far as possible to enable students to participate in the survey quickly. Attention was paid to ensure that the instruments are low-threshold and easy to understand for adolescents with all kinds of educational background. The WHO defines adolescence as the phase from 10 to 19 years of age (World Health Organization, 2019). In our study, adolescents from 13 to 19 years of age were included for the statistical analysis. The present data were collected online. Test subjects were only offered to participate via a link provided by teachers and social media platforms. The survey was available online from April 27th to May 3rd. At the time of data collection, schools in Hamburg and Schleswig-Holstein had already been closed for 6 weeks. There was a ban on contact for more than two people and an order to keep a minimum distance of 1.5 m to other people. An additional obligation to wear mouth-nose covers was introduced in both federal states on April 27th.

#### Physical Activity

Physical activity was measured using the “Godin-Shephard Leisure-Time Physical Activity Questionnaire” (Godin and Shephard, 1985). The GSLTPAQ originally consisted of one item (During a typical 7-day period how many times on the average do you do the following kinds of exercise for more than 15 min during your free time?) and three response categories (strenuous, moderate, mild intensity). The total volume of PA is calculated by metabolic equivalents (METs). The frequency of PA is multiplied by the factor of the PA intensity level, which is three for mild, six for moderate, and nine for strenuous intensities. As mentioned above, German schools were closed for 6 weeks at the time of the data collection. Therefore, the item of the GSLTPAQ was changed to “During a 7-day period how many times on the average do you do the following kinds of activities for more than 15 min?” and thus, the total PA of the students was recorded. Also, the GSLTPAQ is particularly well suited to our intent, because it does not require high-level self-reporting skills (Godin, 2011).

#### FOC-19: (= 0.718)

FOC-19 was measured using the “SARS Fear Scale.” Ho et al. (2005) developed the questionnaire for the Severe Acute Respiratory Syndrome (SARS) discovered in China in 2002 and used it to survey 179 healthcare workers. Based on the sample, the researchers conducted a factor analysis for the items and recommended nine of the 18 items for the further use of the scale. To meet this study’s purpose, six out of nine items were selected, as three of the items were specifically intended for health care workers. Example items include “COVID-19 makes me fear that I will be infected” (factor infection) and “COVID-19 makes me feel that life is threatening” (factor insecurity). Participants responded on a 4-point Likert scale. Answers included “1 = definitely false,” “2 = somewhat false,” “3 = somewhat true,” and “4 = definitely true.”

#### Boredom: (= 0.779)

To measure “state Boredom,” the short form of the “Multidimensional State Boredom Scale” (MSBS-8; Hunter et al., 2016) was chosen. The MSBS was initially published by Fahlman et al. (2011) and consisted of 28 items. The short form is reduced to eight items. The MSBS-8 is an appropriate tool to differentiate between “bored” and “not bored” (Hunter et al., 2016), p246. Compared to other test instruments, the MSBS was designed to measure the current state of boredom and not the boredom proneness. Participants responded to the items on a 7-point Likert scale ranging from “1 = strongly disagree” to “7 = strongly agree.”

#### Covariates

Age, gender, and the federal state of the attended school were collected as demographic covariates.

### Data Analysis

The analysis of the data was performed by using IBM SPSS Statistics 25. The collected data was checked for completeness, exclusion criteria was applied, and the data was checked for outliers. Also, the significance level was set at *p* < 0.05.

### Statistical Analyses

First, the total scores for our three main variables (PA, FOC-19, Boredom) were calculated. The endogenous variable in the regression model was the FOC-19 score. Possible predictors were the overall PA value, the total boredom value, the age, gender and the federal state where the school is located (Hamburg or Schleswig-Holstein). When selecting the regression model, the information criterion according to Akaike (AIC) was used. The model shown in Table 2 has the lowest AIC and will be used for the further study. The Breusch–Pagan test provides no indication of heteroscedasticity, the RESET test does not indicate any incorrect specifications.

## Results

### Descriptive Statistics

Table 1 presents: means, standard deviations, minima and maxima, as well as Pearson correlation coefficients of the variables. The reported FOC-19 significantly correlates with total PA (*r* = −0.214, *p* = 0.017), quantity of strenuous PA (*r* = −0.325, *p* < 0.001), and boredom (*r* = 0.305, *p* < 0.001). Also, of the six FOC-19 items, the item “COVID-19 makes me worry if my family will be infected” has the highest average value with a mean of 3.13 (*SD* = 1.01). “COVID-19 makes me feel very unsafe about myself,” has the lowest average value, with a mean of 1.92 (*SD* = 0.93).

**TABLE 1:** Descriptive statistics and correlation matrix.

*N* = 122	FOC-19	MSBS-8	GSLTPAQ	Strenuous	Moderate	Mild
FOC-19	1					
MSBS-8	0.305**	1				
GSLTPAQ *_(Total)_*	−0.214*	–0.083	1			
Strenuous	−0.325**	−0.211*	0.824**	1		
Moderate	0.058	0.120	0.618**	0.132	1	
Mild	0.016	0.098	0.561**	0.120	0.512**	1
Mean	13.426	33.893	53.868	2.93	3.08	4.04
SD	3.835	8.813	26.707	2.198	2.011	2.494
Minimum	6	8	3	0	0	0
Maximum	23	53	119	10	7	10

*Correlation is significant at the p < 0.05 level (2-tailed); **correlation is significant at the p < 0.01 level (2-tailed); FOC-19, Fear of COVID-19; MSBS-8, Boredom; GSLTPAQ, Physical Activity; SD, Standard Deviation.

### Multiple Regression

Table 2 shows the linear multiple regression model that was calculated to predict FOC-19 based on PA, boredom, and age. A significant regression equation was found [*F*(3,118) = 6.876, *p* < 0.000], with an adjusted *R*^2^ of 0.127. The standardized beta values of PA (= −0.198, *p* = 0.0223) and boredom (= 0.291, *p* ≤ 0.001) were significant. The beta value of the variable age was not significant (= −0.140, *p* = 0.101).

**TABLE 2:** Coefficients and *R*^2^ for the regression model; dependent variable fear of COVID-19.

Variables	Unstandardized	Std. error	Standardized (Beta)	*t*	Sig.
Constant	15.575	3.358		4.638	0.000
GSLTPAQ	−0.028*	0.012	–0.198	–2.315	0.022
MSBS-8	0.127***	0.037	0.291	3.413	0.001
Age	–0.310	0.188	–0.140	–1.648	0.102
*R*^2^	0.149				
adj. *R*^2^	0.127				
*F* (df = 3, 121)	6.876***				

*Significant at p < 0.05 level; ***significant at the p < 0.001 level; GSLTPAQ, Physical Activity; MSBS-8, Boredom.

Participants predicted FOC-19 is equal to 15.575 – 0.028 (PA) + 0.127 (Boredom) – 0.310 (Age). If PA decreases by one unit, the FOC-19 increases by an average of 0.028 units. If boredom increases by one unit, the FOC-19 increases by an average of 0.127 units, and the FOC-19 decreases by an average of 0.310 units with increasing age (years). The three independent variables explain 12.7% of the variance in the dependent variable FOC-19. According to Cohen (1992), this corresponds to a weak to medium size effect.

## Discussion

Higher rates of symptoms of anxiety (6.33–50.9%), depression (14.6–48.3%), post-traumatic stress disorder (7–53.8%), psychological distress (34.43–38%), and stress (8.1–81.9%) were reported by the general population of several countries of the world during the current COVID-19 pandemic (Xiong et al., 2020). According to these authors, to have a younger age, to be a student, and to be frequently exposed to social media/news concerning COVID-19, are, among others, risk factors for suffering any mental problem. Among all of the population groups, adolescents fit the previously mentioned prerequisites, which situates them as a vulnerable group for having a mental problem triggered by the current pandemic.

The present study aimed to investigate the association between PA, boredom, and fear during the COVID-19 pandemic. We have used the naturally occurring circumstances as an opportunity to investigate the mode of action of the variables. The effectiveness of PA (especially PE) as an intervention against anxiety disorders and depression for adolescents and adults, is undeniable in clinical psychology (Ströhle, 2008; Anderson and Shivakumar, 2013; Wegner et al., 2014, 2020; De Souza Moura et al., 2015). Therefore, the question arose whether these effects also take place when a fear stimulus affects otherwise healthy adolescents. The construct boredom was additionally selected because previous research has shown that children and adolescents, in particular, perceive boredom as a severe consequence of isolation (Cava et al., 2005; Brooks et al., 2020).

It was assumed that more bored adolescents have more FOC-19. At the same time, we hypothesized that PA and boredom correlate negatively. However, our study results suggest that the correlation between boredom and PA is small and not significant. Nevertheless, there is a significant positive correlation between boredom and FOC-19; students who have reported more boredom have also reported greater fear of the virus and its consequences. Also, as predicted, there is a significant correlation between PA and FOC-19; students who were physically more active have reported less fear of the virus. Especially the amount of strenuous PA per week shows a significant correlation with the FOC-19, which is in line with the results that exercise intensity matters (Budde et al., 2016b; Gronwald et al., 2018). Also, looking at the individual item scores, the fear of an infection in the family is particularly striking. In addition, the multiple regression model’s calculations have shown that the variables boredom, PA, and Age contribute significantly to predicting the FOC-19.

There are only a few studies that have researched PA during a pandemic, but these show similar results to ours. Maugeri et al. (2020) reported that a reduction of PA during the time of pandemic is associated with poorer mental well-being. In another study on PA and mental health during the COVID-19 pandemic, researchers showed that participants with more PA got higher mental health scores, and participants who became more active during the pandemic had lower anxiety scores (Lesser and Nienhuis, 2020). Besides, it was shown that chronic (regularly conducted) PE shows positive effects on the immune system and can improve the immune response to virus or bacteria (Amatriain-Fernández et al., 2020a). There are various theories of the effectiveness of PA (Wegner et al., 2020). Among others, it is believed that PE induces neuromodulation processes, which can “enhance an individual’s capacity to respond to new demands” (Budde et al., 2020), p2.

In the past, boredom had mainly been identified in qualitative studies. As far as we know, only one study related to state boredom has been published during the COVID-19 pandemic. In their study on adults, Chao et al. (2020) found that state boredom is significantly related to anxiety, stress, and depression, which is in accordance with the data presented here. Still, it should be noted that the researchers did not measure virus-related anxiety, but rather general anxiety symptoms. However, a large number of statements show that, boredom, frustration, and loneliness are perceived as severe consequences of isolation (Ahmed et al., 2020; Kumar and Nayar, 2020; Wang et al., 2020; Xiang et al., 2020).

Our results suggest that adolescents who show both of the indications; less physically active and more bored (during quarantine), show a greater fear of the virus.

According to research data, these effects can decrease with age. Youths are extraordinarily vulnerable to environmental changes, and the foundation for a healthy life must be established for children from 0 to 18 years old (Clark et al., 2020). Thus, clear strategies must be designed to stabilize the conditions that have been thrown off balance in the crisis. For example, measures to maintain (a health contributing amount of) PA could be implemented through schools, and in the case of further lockdowns, online teaching should be top priority. Also, this is important to give students a structure for their daily lives, thereby increase the likelihood of regular PA and sleep (Brazendale et al., 2017). This is significant, as recent research has shown that there is a link between the outbreak of COVID-19 (with the related consequences, such as restrictions on public life, daily media reports, etc.), sleep quality and generalized anxiety (Forte et al., 2020).

However, our research also had several limitations. As the restrictions on public life have changed rapidly, we had to keep the survey period as short as possible. This has influenced the sample size of the study. A larger sample would be desirable for better representativeness and group comparisons.

Also, the fear of COVID-19 questionnaire had to be designed quickly. This limits the standardization of the fear questionnaire, since it was originally made for health care workers and to our knowledge is not able to distinguish between fear and anxiety as desired in research articles (Sylvers et al., 2011). More precise COVID-19 questionnaires are available now, which are validated and are able to survey the fear of COVID-19 more selectively (Ahorsu et al., 2020). Additionally, it’s important to collect more information about teenagers’ parents, socioeconomic status and living conditions, as these could further explain differences in PA. We suspected that any additional question would weaken the quality of the survey, so that this data was not collected. Although the age-specific sample does not allow the extrapolation of the results to the general population, which can be considered a limitation, the specific focus on a vulnerable group of the population, adolescents (Xiong et al., 2020), can also be considered a strength. Since this study is a cross-sectional online study, it is impossible to show any causal relationships as well as to measure any changes in behavior due to the pandemic. An experimental approach to the topic could have enabled this. Another limitation is that PA was measured by self-report questionnaire, since more valid data could have been measured by testing and monitoring the physical fitness and the amount and level of PA supported by reliable devices. The used questionnaire, however, was highly correlated with physical fitness (Jacobs et al., 1993).

In summary, the results found in this study indicate that PA and boredom are significantly correlated to the quarantine experience of adolescent students. Our findings let us assume, that students who are physically more active, and do not feel bored, have less fear of the coronavirus. Future research should use a larger sample to analyze the individual variables in more detail. The factors PA and boredom should be taken into account in the design of quarantine safety measures.

## Data Availability Statement

The datasets presented in this article are not readily available because rights of use were only granted to the authors. Requests to access the datasets should be directed to the corresponding author.

## Ethics Statement

The studies involving human participants were reviewed and approved by the Ethics Commission of the MSH Medical School Hamburg. Written informed consent to participate in this study was provided by the participants or their legal guardian/next of kin.

## Author Contributions

VB and HB contributed to conception and design of the study and performed the statistical analysis. VB organized the database. VB, TG, SA-F, EM-R, SM, and HB wrote the first draft of the manuscript. All authors contributed to the manuscript revision, read, and approved the submitted version.

## Conflict of Interest

The authors declare that the research was conducted in the absence of any commercial or financial relationships that could be construed as a potential conflict of interest.
